# Advancing early and equitable detection of dementia: key learnings/challenges, recent innovations, and future directions

**DOI:** 10.1093/geront/gnaf221

**Published:** 2025-10-01

**Authors:** Joshua Chodosh, Soo Borson, Alexandra Nordyke, Simona C Kwon, Karyn Marsh, Alok Vedvyas, Matthew Lee

**Affiliations:** Division of Geriatric Medicine and Palliative Care, New York University Grossman School of Medicine, New York, New York, United States; VA New York Harbor Healthcare System, New York, New York, United States; Keck School of Medicine, University of Southern California, Los Angeles, California, United States; Department of Psychiatry and Behavioral Sciences, University of Washington School of MedicineWashington, Seattle, United States; Division of Geriatric Medicine and Palliative Care, New York University Grossman School of Medicine, New York, New York, United States; Department of Population Health, New York University Grossman School of Medicine, New York, New York, United States; Department of Neurology, New York University Grossman School of Medicine, New York, New York, United States; Department of Neurology, New York University Grossman School of Medicine, New York, New York, United States; Department of Population Health, New York University Grossman School of Medicine, New York, New York, United States

**Keywords:** Alzheimer’s disease, Dementia, Cognition, Early detection of dementia

## Abstract

Worldwide, over half of all individuals with dementia are undiagnosed. In the United States, racial, ethnic, and economic inequities mirror global findings, with higher rates of missed and delayed diagnosis and poorer diagnostic quality among minoritized and disadvantaged groups. For example, delayed diagnosis is more prevalent among people identifying as non-Hispanic Black or Latino than non-Hispanic White. Systematic efforts to improve detection can increase diagnosis rates; there is broad consensus that earlier detection and initiation of focused care and support services benefit both affected individuals and their loved ones. Systemic under-detection and its contributions to persistent population-level suffering underscore the importance of early detection of dementia as a key public health issue. Improving early detection calls for comprehensive, coordinated responses from local, regional, and national public health systems in partnership with health care delivery systems and community-based organizations. The Public Health Center of Excellence on Early Detection of Dementia (PHCOE on EDD), funded by the Centers for Disease Control and Prevention (CDC), is a national resource to promote understanding and implementation of evidence-based and evidence-informed public health strategy for early detection of dementia. We, together with the PHCOEs on Dementia Risk Reduction and Dementia Caregiving, and nearly four dozen state and local initiatives, seek to operationalize the priorities of the Building Our Largest Dementia Infrastructure for Alzheimer’s Act and National Healthy Brain Initiative, established by federal legislation in 2018 and 2024. Our efforts support the CDC’s mandate to build a national public health infrastructure for brain health and dementia.

A global systematic review and meta-analysis found that dementia was undiagnosed in over 60% of affected individuals (Lang et al., 2017). In the United States, racial, ethnic, and economic inequities mirror global findings, with higher rates of missed and delayed diagnosis and poorer quality of the diagnostic process among minoritized and disadvantaged groups (Cerise, 2021; Tsoy et al., 2021). For example, delayed diagnosis is more prevalent among people identifying as non-Hispanic Black (34.6 months) or Latino (43.8 months) than non-Hispanic White (31.2 months) (Lin et al., 2021). ­Systematic efforts to improve detection can increase diagnosis rates (Borson et al., 2006; Verghese et al., 2024), and there is broad consensus that earlier detection, coupled with focused care and support services, would benefit the well-being of affected individuals and their loved ones. The scale of systemic under-detection, and its contributions to persistent population-level suffering, underscore the importance of early detection of dementia as a key public health issue (Wong et al., 2024).

The Building Our Largest Dementia (BOLD) Infrastructure Public Health Center of Excellence on Early Detection of Dementia (PHCOE on EDD) seeks to promote early detection as a national population health objective through fostering innovation within, and collaboration across, the sectors of public health, health care delivery, and community-based organizations. We define early detection as recognizing and acting on functionally significant cognitive impairment before a crisis occurs. This definition promotes cross-sector understanding, assures practical relevance, and unifies disparate service sectors around a common goal. It is agnostic to setting, designed for broad reach, and consistent with pragmatic and collaborative implementation of programs to provide the right care, at the right time, to the largest number of people, in the most equitable way. In this article, we highlight recent innovations, key takeaways, and future directions on advancing early and equitable detection of dementia across the nation.

## Why early detection of dementia is a public health issue?

Two major features define dementia: (1) a demonstrable decline from previous levels of cognitive ability that is (2) of sufficient severity to compromise everyday function (American Psychiatric Association, 2013). The demand for earlier detection will only grow with the substantial increase in prevalence predicted over the next 40 years. New cases of dementia in America are expected to increase each year, from approximately 514,000 in 2020 to 1 million in 2060 (Fang et al., 2025). Dementia brings with it progressive disability that has broad psychosocial and economic individual-, community-, and population-level impacts. While recent “breakthrough” biomedical therapeutics specific to the very early stages of one cause of dementia—Alzheimer’s disease—have received the lion’s share of public attention, the majority of individuals affected by dementia today will not qualify for or benefit from these treatments. The most pervasive and lasting effect of dementia is the progressive loss of agency, compounded by the many ways family and friends must adapt to meet changing needs for care, while learning to sustain their own self-care. The cost of that adaptation, measured in well-being, health, and dollars, is exacerbated by deficiencies in health and social care systems that are still too poorly organized to deliver timely, comprehensive, collaborative, and longitudinal care management. These costs are compounded by a lack of organizational readiness for dementia care and the myriad risks to the safety of affected individuals, their care partners, and their communities that accompany dementia’s rising prevalence. Meeting these challenges requires clear and ongoing investment in public health.

Early detection is a foundational population health strategy for improving dementia care and outcomes (Blinka et al., 2023). Public health departments, communities, social service agencies, health care delivery systems, and research programs all make specific contributions to repairing the inequitable access to care and widespread gaps in workforce and dementia capability that compromise dementia care quality (Heintz et al., 2020; Jennings et al., 2025; Vasquez et al., 2023; ­Warshaw & Bragg, 2014). Symptomatic of these problems are the persistence of potentially avoidable acute care episodes and the vulnerability to neglect or abuse, accidents and injuries, and social marginalization and stigma that isolate people with dementia and their families. All of these are greatly exacerbated when dementia is unrecognized until after a crisis has occurred. All can be mitigated.

## Early detection of dementia and the 10 essential functions of public health

For over 25 years, Essential Public Health Services (EPHS) have provided a framework for critical actions essential to advancing the mission of public health (Centers for Disease Control and Prevention [CDC], 2020). This framework—originally released in 1994 and updated in 2020—is the result of a collaboration between the Public Health National Center for Innovations (PHNCI) (Public Health Accreditation Board, 2025), the De Beaumont Foundation (2025), and public health experts, leaders, and practitioners (CDC, 2020). We have applied this framework to identify actions that promote early detection of dementia and guide much of what we do in the PHCOE on EDD (see Table 1).

**Table 1.** Ten essential public health services: actions to promote early detection of dementia.

Essential public health service	Example action
**1. Assess and monitor cognitive decline, dementia diagnoses, and dementia care coordination population-wide and across communities**	Use all available data sources that capture cognitive symptoms, diagnoses, and care delivery
**2. Investigate and address gaps in dementia detection efforts**	Identify the best available data sources to help assess population-level detection inequities
**3. Communicate effectively to inform and educate the public on the importance of early detection, and to de-stigmatize dementia and screening**	Develop tailored educational materials to reach communities with messaging on dementia as a biological process with social impact
**4. Strengthen, support, and mobilize communities and partnerships to advance early and equitable dementia detection**	Engage health care systems and community-based organizations to explain the benefits of early detection and promote collective action
**5. Create, champion, and implement policies, plans, and laws that promote early detection of dementia**	Develop and promote policies that provide sustained funding for early detection across sectors
**6. Utilize legal and regulatory actions to encourage early detection of dementia**	Promote benchmarking of Annual Wellness Visit (AWV) implementation; require documentation of how cognitive impairment is identified in AWV
**7. Enable equitable access to early detection**	Identify and promote dementia detection tools validated for the intended population; partner with community-based organizations (CBOs) to promote uptake; facilitate state certification of dementia-capable community health workers (CHWs)
**8. Build a diverse and skilled dementia-capable workforce that understands the importance and value of early detection**	Define team roles and training targets to increase capacity for early detection and transitions to diagnosis and care
**9. Improve and innovate public health action to advance early detection of dementia through evaluation, research, and quality improvement**	Spotlight state and local early detection initiatives and their evaluation metrics
**10. Build and sustain strong agency infrastructure that recognizes dementia detection as relevant to all public health priorities**	Embed brain health and dementia awareness in all public health department sectors, from child health (e.g., grandparents caring for grandchildren) to chronic disease education and monitoring, healthy lifestyle habits training, emergency preparedness, and injury prevention (e.g., transportation safety, falls prevention).

## Why early detection needs a PHCOE?

A public health center of excellence (PHCOE) confers institutional legitimacy, creates opportunities for generative teamwork, sponsors public convenings for reach and impact, and offers economies of scale for dissemination. Dementia—and improving early detection—requires teamwork across three intersecting sectors: public health departments, health care delivery systems, and community-based organizations. The PHCOE on Early Detection of Dementia (EDD) is a vehicle for a concentrated, sustained effort to find and scale solutions to a longstanding problem that is beyond the reach of a single service domain or sector acting alone. Understanding that dementia detection is a multisector activity, we work to ­articulate their intersectionality—areas of existing and potential collaboration. Our success depends on experts and ­champions from three essential domains: clinical content expertise—especially because this has, until recently, been concentrated in specialized academic, medical, and research centers with limited public reach; partnership development, outreach, and convening capacity—core skills of public health departments; and skills in identifying and meeting social needs—core functions of community-based organizations. Underlying this dimensional effort is the expectation that partners will ensure that early detection efforts are grounded in scientific evidence, have broad relevance, and draw from new and existing policy and administrative opportunities. It also requires expertise in program implementation for effectiveness and sustainability. The products of our PHCOE reflect this concentration of effort to increase access to usable, meaningful, and impactful resources.

As a component of the national public health infrastructure, we recognize an obligation to seek and facilitate linkages between existing chronic disease efforts and new or evolving brain health and dementia programs and to translate existing effective chronic disease strategies into dementia-specific programs. Public health’s focus on detection and promoting healthy behaviors has had significant impact on chronic diseases such as hypertension and heart and lung disease. Lessons learned from these efforts provide a strategic platform for presenting dementia as a chronic disease and one that is closely intertwined with other chronic conditions. This perspective leads back to the as-yet unrealized but essential collaboration between public health and health care delivery (Grisham et al., 2023). Our ongoing surveillance of scientific evidence, practice, and policies for closer alignment of clinical and public health efforts aims to inspire actions for useful practice change.

## Focused action of the public health center of excellence on early detection of dementia

To promote early detection of dementia as a routine practice throughout the United States, our PHCOE on EDD has focused on the following primary efforts: (1) to synthesize evidence-based and evidence-informed strategies into written materials that help guide public health, community, and health care systems; (2) to identify and support empowered individuals and stakeholder groups in creating systems for early detection and in seeking to link early detection to comprehensive care; (3) to promote adoption of practices that sustain care over time; (4) to emphasize key opportunities for cross-sector planning; (5) to reduce stigma; (6) to build understanding of dementia as a manageable chronic condition; and (7) to translate research into action steps for stakeholder organizations and communities nationwide. The following sections describe the specific strategies and initiatives that support our Center’s mission.

### Producing written and web-based materials

**Figure 1.**
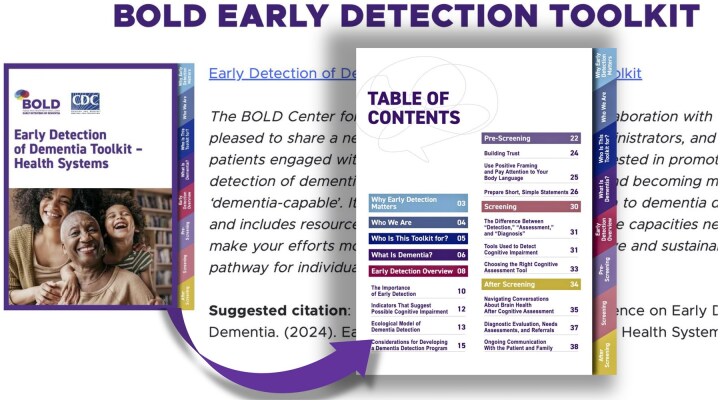
Early detection toolkit for health systems.

Our toolkit development began with a focus on health care and clinical systems (Figure 1), providing individual practitioners and local leaders with strategies for early detection. Our toolkit for health systems provides strategies for effective conversations, messaging to alleviate stigma, and examples of early detection tools, with a supplement providing innovative case examples. More recently, we have focused on developing resource guides for community-based organizations and public health departments with strategies particular to their environments and missions.

Our resource guides focus first on practical ways to reduce stigma and fear, improve understanding of dementia, and promote positive experiences with early detection. We lead with changes in the brain—not the person—as the problem. We present dementia as a chronic disease that, like several others, requires public health intervention, describe its many symptoms and progression as concerns to be actively managed, and seek to normalize brain health conversations as part of routine health care. We provide language that practitioners can use to initiate conversations that encourage agency and realistic optimism. We define the relationship between patient, family, and clinician as a collaborative journey that calls for patience, presence, availability, and continuity.

### Empowering individuals and groups

Our PHCOE-EDD web platform is designed to showcase innovative efforts to initiate, grow, or sustain early detection efforts nationwide. Understanding that not all success stories are linear or smooth, we include projects from early to advanced phases of development and encourage open dialogue about failures and successes. Our webinar series similarly features lessons learned and key takeaways from emerging and established leaders engaged in promoting earlier detection as a pathway to better care and outcomes.

**Figure 2.**
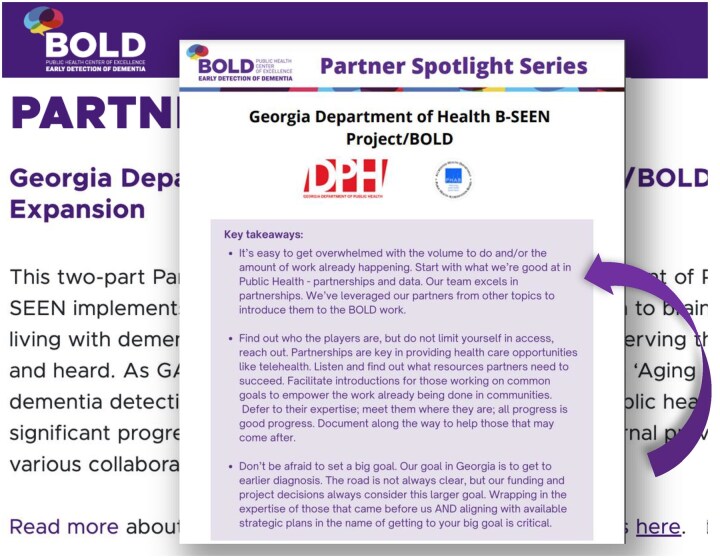
Partner spotlight (PHCOE EDD website).

We work to communicate a unified message and simplify processes that combine scientific evidence and clinical expertise. We prioritize a participatory approach to developing materials, including resource guides, partner spotlights (Figure 2), webinars, and other resources. We convened a National Advisory Council to ensure broad stakeholder representation: state DOHs, community-based organizations (CBOs), ADRD and older adult advocacy groups, specialists in health care and community support for aging rural and Native American populations, health system leaders and clinicians, clinical educators, and Geriatric Workforce Enhancement Program (GWEP) leaders. Our National Advisory Council members, organized into three sector-specific workgroups, co-lead the process of developing, generating, and disseminating products. This process also includes member-checking with end-users to ensure that products are clear and useful.

### Embedding early detection in a population health management strategy

Like heart disease, lung disease, kidney disease, and cancer, dementia is a chronic condition that requires long-range collaboration among medical care, community-based support, and public health organizations. The chronic disease perspective demands an understanding of dementia as more expansive than that encompassed by purely biological definitions. The emergence of specific tests for Alzheimer’s disease pathobiology (and progress toward the same goal in other dementias) is a stimulus for identifying individuals in very early stages of cognitive decline or even before symptoms develop, while a public health perspective on early detection focuses on people with dementia as a definable population with certain shared characteristics, risks, needs, and health impacts regardless of the underlying biology. Focusing this way on dementia as a population health management opportunity, a primary purpose of early detection is to work toward optimal outcomes and prevent, reduce, or mitigate crises for individuals, families, and communities. Proactive, comprehensive, and sustained care requires a cross-sector approach.

### Articulating the shared “why” of early detection: centering crisis risk reduction, mitigation, and management as foci for cross sector planning

A focus on understanding crises in dementia—events that “shock” or disrupt the ongoing pattern of life for individuals and families—brings a unifying focus on outcomes that is easily communicated across sectors. Crisis events commonly arise through behavioral or medical decompensation, accidental injury, caregiver exhaustion, or a combination of these as dementia progresses and individuals age into states of compromised resilience. Crises are also a common cause of permanent transfer from home to long-term residential care and a major source of dementia-related public spending (Choi et al., 2023; Coe, et al., 2023). Extensive research shows that dementia detection tends to be delayed, at the population level, into moderate and severe stages of cognitive decline when crises are most likely to occur. More recently, a study using Medicare claims shows that the great majority of diagnoses occur *in the midst of an existing crisis*, evidenced by a large spike in aggregated health care use (emergency, hospital, and ambulatory care visits) around the time a first dementia diagnosis code appears in the medical record (Jacobson et al., 2024).

If public health, health care, and communities came together to share responsibility for dementia detection and surveillance, early detection could become part of a crisis or emergency prevention and preparedness strategy. Older adults with cognitive and physical impairments are known to be especially vulnerable to injury, and in emergency situations—such as rapidly spreading fires, civil unrest, or severe weather events—people with dementia, who often cannot recognize and respond effectively to danger on their own, may be overlooked unless rescue workers know how to find them. This perspective places early detection and dementia awareness squarely within the public health spheres of emergency preparedness and injury prevention. Both call for the development of comprehensive and coordinated responses from public health systems locally and nationally to develop, implement, and sustain dementia detection approaches and embed them in public safety planning.

### Confronting stigma

Despite rising awareness and new efforts to normalize conversations around dementia, detection efforts continue to be challenged by stigma and compounded by ageism. Stigma—an exclusionary social ill—is found in most communities (though the details may differ), but also among clinicians, health systems, and others called upon to help and support individuals impaired by dementia. Stigmatization of people with dementia arises from both the primary effects of the condition on personal agency, sense of loss, and adherence to social norms but also from fear, unwarranted catastrophic expectations, and lack of understanding. A core tactic in reducing stigma is disseminating knowledge: that dementia results from disease and/or injury to the brain; that it is associated with aging but not present in most older adults; and that it is a chronic medical condition, analogous to heart, lung, and kidney disease, which are also age-related chronic conditions but carry no particular stigma. The stigma around dementia is found in institutions as well as within individuals and communities; when stigma is institutionalized and compounded by ageism, it perpetuates inattention to and outright avoidance of planning and preparation. This results in failure to develop and spread care approaches that make dementia manageable (Lopez et al., 2020). Stigma contributes to the perpetuation of ignorance about dementia, and limited understanding and inexperience make potentially difficult conversations even more so and promote the cycle of avoidance. We advocate for normalizing conversations within and across these multisector groups. The essential elements of constructive conversations can be modeled, taught, and learned. We are invested in co-creating ­strategies to achieve this goal with individuals, ­communities, ­clinicians, health care systems, and public health organizations.

### Chronic care: systematizing approaches to long-range management of dementia

Because dementia is almost always a chronic condition that progresses over years—usually accompanied by other chronic conditions requiring intervention—long-term, whole-person management is a key focus for future public health strategies in partnership with health and community care systems. Implementing appropriate proactive, supportive interventions that are updated and sustained over time requires engaged stakeholders in public health, community organizations, and health care systems. These call for effective public messaging, health and social care policies, payment mechanisms, and organizational infrastructure that embrace dementia detection and care as a population health management concern. Our Center recognizes that early, population-based detection of dementia is a critical step toward justifying the need for, and systematizing such care, for which a solid evidence base is accumulating step by step. Linking detection to care is a focus for ongoing development of Center activities.

### Technical assistance: translating good ideas into successful practice

We promote our capacity for technical assistance (TA) to community-based organizations, BOLD-funded (and non-BOLD) public health departments, health care system working groups, and others. We emphasize the value of defining the impact our partners want to have; this powerful question, often surprisingly difficult to answer, has proven particularly helpful in focusing our TA efforts. We use broad communication strategies, including in-person and virtual events and web postings, to promote our technical assistance, which is focused on building capacity for key partners nationally, identifying feasible implementation steps, and planning strategies for maximal impact. This includes practical applications that build on the Center’s implementation expertise and the science underpinning it.

**Figure 3.**
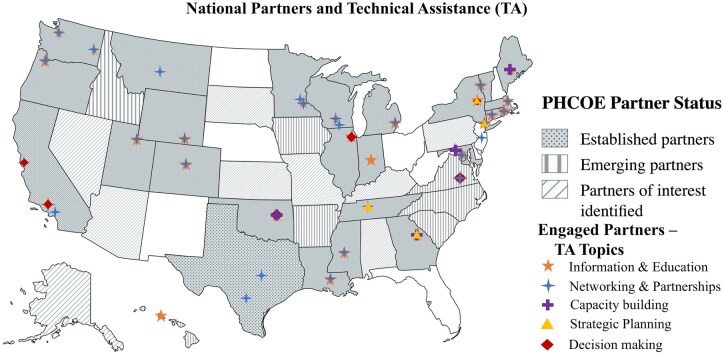
National partner status and technical assistance (TA) topics.

We tailor TA to meet the specific needs of each program, its target communities, and its capacity for implementing new strategies. Topics often fall within these five activities: (1) information and education; (2) networking and partnerships; (3) capacity building; (4) strategic planning; and (5) decision making (Table 2; Figure 3). As in all our initiatives, we work to amplify the need to integrate detection activities into primary care and other health care sectors, public health programs, and community organizations serving older adults. Table 3 outlines examples of specific TA we provide to our national partners.

**Table 2.** Technical assistance topic areas.

Technical assistance topics	Program support
**Information and education**	Share information and publications from emerging research. Identify a screening approach most likely to be accepted based on local culture and attitudes. Tailor conversations and presentations for specific communities.
**Decision-making**	Program planning: initiatives, logic models, and pilot studies. Careful considerations to support PCPs in their assessments and referral mechanisms and to tailor DOH-led community education.
**Capacity building**	Further develop existing resources to inform and lead community education events. Review BOLD program products, PSA campaigns, and resources. Identify key considerations for evaluation.
**Networking and partnership**	Connect emerging organizations and programs to established programs and training materials. Facilitate BOLD-related collaboration nationwide.

*Note.* PCP = primary care physician; DOH = Department of Health; BOLD = Building Our Largest Dementia Infrastructure; PSA = public service announcement.

**Table 3.** Program-specific technical assistance provided by PHCOE on EDD.

BOLD program(s)	Program-specific support
**Northeast BOLD collaborative (CT, RI, ME, VT, Boston, NY, NYC)**	Pragmatic approaches to clinical-community linkages, state- and local-level data acquisition, registry, and monitoring systems.
**Oklahoma BOLD Program**	How to talk about cognition where there are cultural barriers.
**Maine BOLD Program**	Drilling down on community-level data to identify groups with high prevalence and low resources in the state. HealthReach FQHC, Maine BOLD, and PHCOE on EDD partnered to co-develop and provide trainings to FQHC network. Training topics included: how to have pre- and post-screening conversations, how to initiate a care plan with three patient cases, and how to make a diagnosis.
**Georgia BOLD Program**	Provided evidence about implementing cognitive screening in public health clinics. Reviewed early workflow development plan. Provided advice on defining impact for their screen-to-diagnosis program.
**Los Angeles County BOLD Program**	Reviewed early detection workplan. Assisted in planning health system summit. Co-leading early detection implementation workgroup.

*Note.* BOLD = Building Our Largest Dementia Infrastructure; FQHC = Federally Qualified Health Center; PHCOE = Public Health Center of Excellence; EDD = early detection of dementia.

## Public health tools for early detection

**Figure 4.**
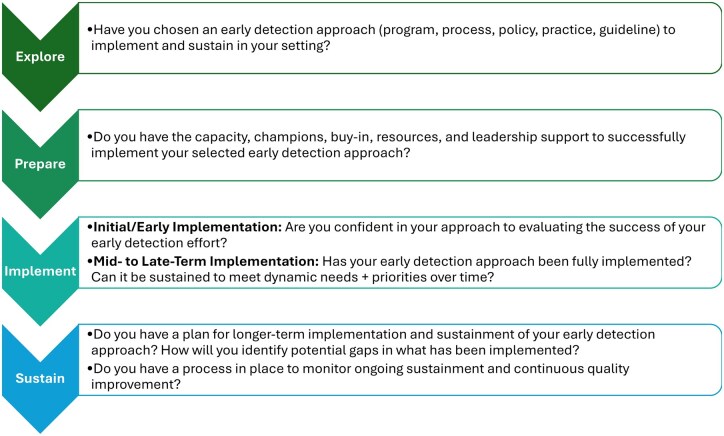
Abbreviated tool: implementing and sustaining change to advance early dementia detection.

We are often asked to recommend the best tool for early detection of dementia. There is no one “best” tool, and not even a single best category of tools: there are many, and they do best when selected for a specific population and setting, and with a specific purpose and goal. Tools for public health include cognitive screening tools—the common meaning of “tools for early detection”—but other kinds of tools are equally or more important. They include evidence-based resources for educating the public about the importance of dementia detection and ways to educate state and local health officials about the consequences of ignoring dementia and how to overcome this barrier. The health care system is usually considered “the place” for dementia detection; the Centers for Medicare and Medicaid Services (CMS) provides a range of benefits that reimburse clinicians and health care systems for specific dementia detection, diagnosis, and management activities. CMS benefits are themselves tools for health care systems; however, they are permissive but rarely prescriptive. That is, they provide a means for willing clinicians and health systems to take on early detection and systematic care—but there is no requirement to do so. The exception, initiated in January 2025, is the new CMS requirement that hospitals report an “age-friendly hospitals” measure (including impaired mentation) based on the Institute for Healthcare Improvement’s Age-Friendly Health System framework (The John A. Hartford Foundation, 2024; Institute for Healthcare Improvement [IHI], n.d.). There is no consistent motivation or standardized approach to achieving such a goal within other sectors of the U.S. health care system, including primary care—the part of the health system most capable of improving population health (Starfield et al., 2005). This creates significant opportunities for public health and community organizations to craft their own early detection programs and consult and collaborate with health care partners to design and implement workable, locally-tailored strategies to meet the goals of all three sectors. Some tools that support effective detection strategies include population needs assessments and risk stratification algorithms, implementation and sustainability tools (see Figure 4 below for an abbreviated version of a new tool available on our Center website [Aarons et al., 2011; Moullin et al., 2019]), and clinical and community-based staff training in difficult conversations, formal cognitive impairment screening tests, diagnostic algorithms, and workflows designed to generate feasible and effective care plans.

## Expanding workforce roles for dementia detection and care

The three PHCOEs (Risk Reduction, Early Detection, and Caregiving) are working together to delineate dementia core competencies for Community Health Workers (CHWs) and partner with other public agencies in curriculum development. While CHWs are not yet formally certified in many states, they can fill essential roles in all three sectors where dementia care is a focus. Medicaid can reimburse their work, and as of 2024, so can Medicare, provided CHWs work in a suitable setting and their role is recognized in the state where they work. In addition, we contribute to defining new dementia care roles and curricula for Registered Nurses (University of Washington and Yale Schools of Nursing, n.d.).

## Identifying data resources for progress in early detection

Public health tools include targeted data collection to assess a population’s baseline characteristics and the impact of educational campaigns, practice innovations, and workforce development initiatives. Surveys, a widely used form of data collection for public health, need to be combined with data generated from other sectors, especially health care delivery and community service and support systems, county, state, and federal health administrative units, and funded research.

### Behavioral risk factor surveillance system

Public health data specific to dementia detection is exemplified by the Behavioral Risk Factor Surveillance System (BRFSS), a cross-sectional telephone self-report survey conducted by all 50 states and three territories on chronic health conditions and health behaviors. First created by CDC in 1984, BRFSS is a collaborative effort between CDC and state and territory health departments and includes three sets of questions: core questions that each state and territory must ask, optional modules, and questions that a state or territory adds based on their identified health priorities. The BRFSS optional module on cognitive decline is a 5-item measure that queries an individual’s self-perceived cognitive decline, known as subjective cognitive decline. Most relevant to the PHCOE on EDD is the third question in that module, “Have you or anyone else discussed your difficulties with thinking or memory with a health care provider?” For about half of individuals reporting subjective cognitive decline, the answer is “no.” Interpretation of these results is impacted by low response rates and inconsistent repeat deployment of the module, limiting its usefulness as an index of progress in early detection. As with most initiatives implemented at the state level, it is uncertain how well BRFSS data reflect the impact of public health initiatives. A BRFSS sampling approach that allows for incorporating data from health and community care would enrich the policy opportunities inherent in state-level telephone surveys for public health action. During one of our more recent meetings with a collaborative of northeast BOLD programs (Maine, Massachusetts, Rhode Island, Vermont, Connecticut, and New York), we initiated plans to coordinate the deployment of the BRFSS Cognitive Decline Module and will continue to develop strategies for broader regional impact on public health and healthcare policy.

### Federal health care data sources

Important potential measurement opportunities are found in the health care sector through federal and state insurance programs (Medicare, Medicaid, and programs in medically underserved areas [e.g., Federally Qualified Health Centers, Community Clinics, and Rural Health Clinics]), and from community-delivered health programs, such as home health agency services that use standardized Outcome and Assessment Information Set (OASIS) data. Cognitive data from OASIS can be associated with in-survey diagnostic data. As with insurance reimbursement data, utility is limited by access to specific ­services with less usefulness among underserved and under-represented groups.

### Electronic health records

Electronic health record (EHR) data are widely used to answer health risk and care delivery questions in health services research but are historically inaccessible to public health agencies. Data sharing enabled by appropriate privacy and proprietary protections could further leverage public health evaluation and impact. An increasing number of purpose-built EHR data registries offer new opportunities, and uniform software systems could improve generalizability: for example, a single EHR system—Epic—is now used by over 80% of health care systems in the United States (Eaquinto, 2023). However, current standards of care do not require that health systems implement practice management tools such as dementia registries, detection activities benchmarked to expected prevalence rates, templates for structured clinical evaluations, use of common measures, or easily identified care plans that could generate useful data.

### Need for standards of practice to drive data for evaluation

Although many professional organizations (e.g., the American Academy of Family Practice and the Gerontological Advanced Practice Nurses Association) offer high-quality, accessible educational materials and guidance for members, clinicians are not required to meet a uniform expected national standard of practice for dementia detection and care. Thus far, efforts to mount a “business case” have had little effect on health care systems, although some local efforts are proceeding through both proprietary and/or funded research programs; there is at least one health care system that provides direct salary enhancements for clinicians who meet or exceed a defined proportion of Annual Wellness Visits—a Medicare benefit that includes “detection of any cognitive impairment” as an element required for reimbursement. Moreover, as of January 2025, CMS has taken the decisive step to require hospitals to report a new quality measure, attesting to their adoption of “Age Friendly” Health System principles and practices (including identification of cognitive impairment) among hospitalized patients. This requirement is likely to have, over time, some influence on other sectors of health care delivery, including ambulatory care (IHI, 2024).

The as-yet-unrealized value of standardizing practice and coding dementia-relevant process measures is a natural focus of the PHCOE on EDD. In addition to organizing, participating in, and following up on local, regional, and national professional and cross-sector meetings, our efforts include ongoing surveillance of the federal landscape (e.g., identifying new Medicare benefits relevant to dementia detection and care, tracking the progress of model demonstrations such as GUIDE [CMS, 2024]). We attend as many presentations as possible by national funders and developers (e.g., CMS, Agency for Healthcare Research and Quality [AHRQ], and Health Resources and Services Administration [HRSA]) in search of pragmatic opportunities to insert measures that capture dementia-relevant activities within ongoing health care initiatives.

## Leveraging clinical training to support public health impact

### Disseminating national training for practitioners

The PHCOE on EDD has initiated conversations with recipients of HRSA’s Geriatric Workforce Enhancement Program (GWEP) to broaden access to dementia-specific training. GWEP programs reach diverse health care disciplines with didactic and case-based learning to help attendees recognize signs of cognitive impairment, distinguish dementia from depression and delirium, practice skills in communicating a diagnosis and developing a care plan, and evaluate medication use in the context of dementia. In addition, through our website, we help to disseminate locally developed trainings for clinicians in dementia detection, including the University of Washington’s Cognition in Primary Care Program (University of Washington, n.d.) and Dementia Care Aware in California, both of which have received wide recognition.

### Training clinicians for dementia detection in non-Medicare populations

Older adults who do not qualify for Medicare (e.g., are not citizens or legal residents, or do not qualify for Social Security benefits) have no access to the Medicare Annual Wellness Visit as a vehicle for detecting cognitive impairment. Dementia Care Aware (DCA, 2025) is a unique training program that supports dementia training for primary care clinicians caring for older adults in any setting, but with special emphasis on Medicaid-only populations. DCA’s web-based training provides relevant information about dementia and simple detection and management tools that any clinician can use; those practicing in California can use a unique “Cognitive Health Assessment” (CHA) billing code under Medi-Cal (California’s Medicaid plan).

## Future strategies to gain traction and accelerate impact

As we conclude five years of funding, we reflect on the collaborations we have built, the resources we have created, and the momentum we have helped generate, but any achievements are a small fraction of what we hope to accomplish. We have disseminated the successes of “dementia activists” and promoted the talents of those implementers. However, with every success, we note the uniqueness of the environment in which it occurred and the challenges to spread and generalization. Moreover, amplifying the success of others, while an essential focus of our work, is a passive process that does not guarantee suitability for or adoption in other settings. To further promote change, we will focus on identifying communities and organizations with the greatest need and, specifically, interventions within those organizations or component parts that may be scalable. One area of attention will be on Federally Qualified Health Centers, which provide community-based, accessible primary care to over 30 million low-income and uninsured patients annually (Horstman et al., 2024, Lee et al., 2023). Their community focus, national platform, unique funding structure, mandated biopsychosocial approach to care, opportunities to engage multiple stakeholders, and culture of inclusivity could provide special opportunities to develop a unified approach to dementia detection and care as part of the organic evolution of cross-sector integration.

We have had many meetings with stakeholder groups that include only one sector, perpetuating the silos we aim to breach. To address the challenge of creating structural collaborations between health care, community, and public health organizations, we intend to engage stakeholders with the explicit expectation of alignment with cross-sector interdependence as both a value and as a fact. We will identify and convene meetings of dementia detection and care champions in each sector to create a new collaborative strategy, fostered by representative participation and oriented toward consolidation of interprofessional goal-setting. Such an approach respects both the sector-specific operations, objectives, and achievements and their inevitable interconnectedness and will promote methods for combining data across interlinked systems that will better serve population health and care needs.

Finally, because we remain steadfast in our belief that dementia detection and care will be most impactful when explicitly embraced within a primary care framework, we will convene focus groups of clinicians who will be charged with finding solutions to more effective delivery. We will bring what we learn to new convenings of public health departments and community organizations, with the aim of consolidating cross-sector collaboration into a practical blueprint for spreading of best practices in dementia detection and care.

## Summary

Effective education, outreach, and screening have improved surveillance and care for several chronic conditions, including cancer, hypertension, and diabetes, and promoted health behaviors aimed at reducing risk (Ravenell et al., 2023). In the PHCOE on EDD, we develop and help disseminate pragmatic approaches to advancing early detection of dementia, embedding dementia awareness within public health, community, and health care organizations and transforming stigma into understanding of dementia as a manageable chronic condition. In this paper, we have provided an overview of our goals, objectives, methods, and products and outlined practical strategies for partnership across public health, health care delivery, and community service sectors. We highlight some of the opportunities, impediments, and indicators of progress toward improving early detection. We argue for the value of clarifying the “why” of early detection and collaborating across sectors to promote collective impact. Finally, we describe opportunities for further development of tools, strategies, programs, and data systems centered on the common goal of better brain health and better care for aging populations at risk and affected by dementia.

## Data Availability

This article does not report data and therefore the pre-registration and data availability requirements are not applicable.
